# Economic impact of medical genetic testing on clinical applications in Thailand

**DOI:** 10.1371/journal.pone.0243934

**Published:** 2020-12-18

**Authors:** Jiraphun Jittikoon, Sermsiri Sangroongruangsri, Montarat Thavorncharoensap, Natthakan Chitpim, Usa Chaikledkaew

**Affiliations:** 1 Department of Biochemistry, Faculty of Pharmacy, Mahidol University, Bangkok, Thailand; 2 Social Administrative Pharmacy Division, Department of Pharmacy, Faculty of Pharmacy, Mahidol University, Bangkok, Thailand; 3 Mahidol University Health Technology Assessment (MUHTA) Graduate Program, Mahidol University, Bangkok, Thailand; 4 Social, Economic and Administrative Pharmacy (SEAP) Graduate Program, Department of Pharmacy, Faculty of Pharmacy, Mahidol University, Bangkok, Thailand; Edith Cowan University, AUSTRALIA

## Abstract

**Background:**

Although the clinical benefits of medical genetic testing have been proven, there has been limited evidence on its economic impact in Thai setting. Thus, this study aimed to evaluate the economic impact of genetic testing services provided by the Center for Medical Genomics (CMG) in Thailand.

**Methods:**

Cost-benefit analysis was conducted from provider and societal perspectives. Cost and output data of genetic testing services provided by the CMG during 2014 to 2018 and published literature reviews were applied to estimate the costs and benefits. Monetary benefits related to genetic testing services were derived through human capital approach.

**Results:**

The total operation cost was 126 million baht over five years with an average annual cost of 21 million baht per year. The net benefit, benefit-to-cost ratio, and return on investment were 5,477 million baht, 43 times, and 42 times, respectively. Productivity gain was the highest proportion (50.57%) of the total benefit.

**Conclusions:**

The provision of genetic testing services at the CMG gained much more benefits than the cost. This study highlighted a good value for money in the establishment of medical genomics settings in Thailand and other developing countries.

## Introduction

Medical genetic testing is usually used to examine any changes of the chromosomes and genes, which could provide important data for doctors to determine a patient’s chance of developing severe adverse drug reactions (ADRs), the failure of treatment, or a genetic disorder [1]. Such data could also help doctors adjust medication regimens to prevent ineffective treatments and serious ADRs, predict genetic disorders, or provide information if patients might be at risk to develop diseases related to gene mutation [2]. This would lead to a great benefit for preventing morbidities and mortalities for a number of diseases through early detection and effective intervention [3]. Although medical genetic testing is widely available in developed countries, access to genetic testing services for medical proposes in developing countries is commonly restricted to only the wealthy people who can afford such expensive services [4]. According to the World Health Organization (WHO), medical genetic testing should be introduced, publicly funded and overseen by the government to support equitable access and assure the quality of testing in developing countries [4]. Lesson learned from successful, existing genomic centers in developing countries have demonstrated that there were many barriers that impede the development of genetic medicine, such as the availability of sufficient research funding, well-trained programs for medical genetic personnel, national regulatory systems, and effective health policies. To overcome these bottle-neck situations and facilitate the development of medical genomic research and services, local infrastructures and the ecology of medical care for medical genetic research and services should be urgently established and improved [5, 6].

In 2005, the Thai Pharmacogenomics Project was initiated by the Faculty of Medicine, Ramathibodi Hospital with partial support from the Thailand Center of Excellence for Life Science (TCELS) to identify the genetics that could affect drug response, metabolism, ADR, and toxicity on certain key diseases [7, 8]. Later in 2016, the project was renamed as the Center for Medical Genomics (CMG) serving as one of the five governmental medical genetic testing services for the Thai population with next generation sequencing (NGS) technology. Until recently, the CMG has provided genetic testing services for HIV drug resistance using NGS, preimplantation genetic diagnosis for aneuploidies, BRCA1/2 testing using NGS technology for breast and ovarian cancer risk prediction, noninvasive prenatal testing (NIPT), and hereditary cardiomyopathy panel testing using NGS technology [9]. Whole exome and genome sequencing were also performed for research purposes at the CMG.

Based on the viewpoint of the CMG as a provider, providing medical genetic testing services not only generated their income, but was also cost-saving in the country by avoiding sending examinations abroad which charged a higher fee. Moreover, according to the perspective of people who received genetic testing with positive results, the services can help prevent morbidities and mortalities due to early detection and effective intervention. This leads to increased economic benefits, in terms of cost avoidance from healthcare utilization in the future and income earnings or productive gain owing to longer survival. In line with the WHO’s recommendation, there is a significant need to generate economic evaluation evidence on medical genetic testing services to support investments in establishing medical genomics settings towards promoting a more cost-effective health care system [10], since such services are still underutilized to reduce the burden of disease in most developing countries, including Thailand [4]. However, no study has investigated yet the economic impact of medical genetic services in Thailand. Therefore, the objective of this study was to assess the costs and benefits of genetic testing services provided by the CMG during 2014 to 2018 based on provider and societal perspectives to provide suggestions for policy makers whether the establishment of CMG would be a good value for money in Thailand. This information will be valuable for policy decision-making on the provision of medical genetic testing services not only in Thailand, but also in other developing countries.

## Materials and methods

Cost-benefit analysis (CBA) was used to estimate the costs and benefits after the provision of medical genetic testing services by the CMG in Thailand based on a societal perspective, which considers all costs and benefits incurred by everyone in the society according to the Thai Health Technology Assessment (HTA) guidelines [11]. Moreover, an analysis from the provider perspective that included only the CMG’s revenue was performed. The costs of the establishment and operation of the CMG during the year 2014 to 2018 were obtained through primary data collection from the CMG. Both direct and indirect benefits were included to estimate the total benefits. Data used to calculate the benefits were retrieved from the CMG and published literatures in Thailand and from international countries. We searched variables for calculating cost avoidance and productivity gain (S2 Table) from PubMed and Google search engines until December 2018. We applied search terms about probabilities, costs, and life expectancies of patients with diseases related to genetic testing in both Thai and English languages. Then, the quality of data obtained from these literatures was assessed and verified by experts and physicians to ensure reliability and generalizability to the Thai context. The ethical approval for this study was granted by an institutional review board at the Faculty of Medicine, Ramathibodi Hospital. Since the study involved analysis of secondary data, which could not be linked to the subject’s identity, a written informed consent was waived by the ethics committee. The CBA results were presented in terms of: (1) net benefits calculated by the difference between the total benefit and the total cost; (2) benefit-to-cost ratio calculated by total benefit divided by total cost; and, (3) return on investment calculated by net benefit divided by total cost. All costs and benefits were adjusted to present values in the year 2018 using the Consumer Price Index [12].

### Direct benefits

Direct benefits included: (1) revenue generated from genetic testing services calculated by the total number of each service multiplied by its unit price obtained from the CMG; and, (2) cost-saving from genetic testing services at the CMG calculated by the difference between the costs of genetic testing provided by genetic testing agencies overseas, such as the United States, United Kingdom or Canada, and those provided by the CMG in Thailand. The prices of genetic testing services performed by agencies overseas were obtained through literature reviews and available price list from their agencies’ websites. These were adjusted to Thai baht using an exchange rate referred from the Bank of Thailand (1 US dollar = 33.06 baht, 1 Canadian dollar = 25.11 baht and 1 pound = 43.19 baht) [13]. The S1 Table shows the price of genetic testing services at the CMG and the agencies overseas.

### Indirect benefits

Indirect benefits included cost avoidance and productivity gain due to genetic testing. Cost avoidance, which is the expected cost of treatment for diseases or conditions that could be avoided by receiving genetic testing services, was calculated by the number of people receiving genetic testing with positive results multiplied by the cost of treatment for diseases, which might occur in the future per case. We assumed that they received medical care and treatment throughout their lifetime period. Therefore, future costs incurred more than one year were adjusted using a discount rate of 3% based on the Thai HTA guidelines [11]. Moreover, genetic testing can help prevent death or extend life expectancy, which implied that they can gain more productivity to society. Productivity gain was estimated using a human capital approach, i.e., the number of life-years that are expected to increase when comparing between people with genetic testing services and those without as derived from published economic evaluation studies multiplied by the expected income per year using an income growth rate at 5% and a discount rate at 3% [11]. To calculate productivity gain, the following formula is presented, such that L is the increased productivity value, Y_t_ is the expected income to be received in year t, r is the discount rate, and t is the number of years expected to live without death:

$$L=\sum_{t=t}^{T}Y_{t}{\left(1+r\right)}^{-(T-t)}$$

Since genetic testing can help prevent different types of diseases or conditions that are predicted to be avoided in the future, cost avoidance and productivity gain for each genetic testing were calculated except for whole exome and genome sequencing as they are still being researched and have not been disclosed to the public. Table 1 presents the types of benefit included for each genetic testing. All parameters used were validated with clinical experts to ensure reliability of data. The S2 Table presents all parameter values used to calculate cost avoidance and productivity gain.

**Table 1:** Types of benefit included for each genetic testing.

Medical genetic testing services	Revenues	Cost saving	Cost avoidance	Productivity gain
**HIV drug resistance using Next Generation Sequencing (NGS)**				
HIV drug resistance	✔	✔	✔	✔
**Preimplantation genetic diagnosis for aneuploidies**				
Multiplex SNP genotyping	✔	✔	✔	
Low pass whole genome sequencing—WGA				
Mitochondrial DNA analysis–Encephalomyopathy				
Prenatal diagnosis using direct mutation analysis				
**BRCA1/2 using NGS**				
HBOC panel testing–blood by NGS	✔	✔	✔	✔
**Whole exome and genome sequencing**				
Whole gene mutation screening–Others by NGS	✔	✔		
Multiple coding region sequencing by NGS	✔	✔		
Targeted gene sequencing analysis by NGS	✔	✔		
Whole gene sequencing–Others	✔	✔		
Next generation viral sequencing	✔	✔		
**Non Invasive Prenatal Testing (NIPT) using NGS**				
IONA NIPT by NGS	✔	✔	✔	
Thai NIPT by NGS				
**Hereditary cardiomyopathy panel testing using NGS technology**				
Hereditary cardiomyopathy panel testing by NGS	✔	✔	✔	✔

#### HIV drug resistance using NGS technology

When people living with HIV have poor response to antiretroviral (ARV) drugs or do not respond to them, doctors have two options: (1) use second-line ARV drugs; or, (2) test for HIV drug resistance to help select appropriate drugs. If HIV-resistant genes or positive test results were found, the doctor would have to change to second-line ARV drugs to prevent treatment failure and the spread of drug resistance in the community and country levels. If negative test results were found, the doctor could still prescribe first-line ARV drugs, which are cheaper as compared with the second-line drugs. Since the first-line ARV therapy is cheaper than the second-line ARV therapy for patients who do not have HIV-resistant genes, this can reduce the cost of treatment. Therefore, cost avoidance can be calculated by the difference in treatment costs for all patients changing to the second-line ARV drugs and those for patients with negative test results receiving the first-line drugs. To calculate the treatment costs, we assumed that the first-line ARV therapy was the combination of tenofovir (TDF) 300 mg, emtricitabine (FTC) 200 mg, and efavirenz (EFV) 600 mg every 24 hours. Meanwhile, the second-line ARV therapy consisted of TDF 300 mg every 24 hours, zidovudine (AZT) 300 mg every 12 hours, and lopinavir/ritonavir (LPV/r) 400/100 mg every 12 hours based on the guidelines for the diagnosis and treatment of people with HIV and AIDS [14] and experts’ opinion. We also assumed that the patients would use the same type of ARV drugs and have good adherence throughout their lifetime period. We assumed that patients in both options consumed the same healthcare resources and had the same cost in managing side effects due to ARV drugs. Furthermore, genetic testing for HIV drug resistance can help extend HIV patients’ survival as they can receive appropriate ARV treatment after genetic testing. Therefore, productivity gain was calculated as the product of the number of years gained for those receiving genetic testing for HIV drug resistance as compared with those without and annual income per person.

#### Preimplantation genetic testing for aneuploidies using NGS technology

Screening the embryos for chromosomal abnormalities or genetic diseases will increase the chances of successful pregnancies. The genetic diseases that can be avoided include beta-thalassemia, Gaucher's syndrome, DiGeorge-Velocardiofacial syndrome, polycystic kidney disease, neurofibromatosis type 1, hemophilia B, spinal muscular atrophy, Duchenne muscular dystrophy, Marfan syndrome, glutaric acidemia IIB, osteogenesis imperfecta types I–IV, Hallervorden-Spatz syndrome, Ellis-van Creveld syndrome, and multiple congenital anomalies-hypotonia-seizures syndrome 2, etc. Screening chromosomal abnormalities or genetic diseases before implantation in the uterus can prevent the recurrence of genetic diseases in a family whose first child has a genetic disease. It can also reduce the burden of medical expenses on the care for patients with genetic diseases and the psychological impact on mothers when deciding to terminate pregnancies, as well as increase the chances of having children in families who are ready to have children. We assumed that the treatment for genetic diseases would be provided to patients for the rest of their lives. Cost avoidance was then calculated by annual treatment cost multiplied by a patient’s survival for each genetic disease. However, there was no productivity gain from preimplantation genetic testing.

#### BRCA1/2 using NGS technology

Screening for BRCA1/2 genes to detect mutations in breast cancer and ovarian cancer can help inform the risk of family members who are considered to be at high risk for developing breast and ovarian cancer. Therefore, those at high risk would have the opportunity to enter the disease surveillance program, such as mammogram for genetic breast cancer, resulting in reducing the risk of death from the early detection of the disease and avoid the cost of treatment for breast and ovarian cancer at an advanced stage. Cost avoidance was calculated by the treatment cost of breast and ovarian cancer at an advanced stage which could be avoided in the future. We assumed that the treatment for patients with breast and ovarian cancer at an advanced stage would be given throughout their lifetime period. Productivity gain was calculated by the annual income of patients multiplied by the number of life months gained for those with BRCA1/2 testing compared to those without.

#### Noninvasive Prenatal Testing (NIPT)

Noninvasive Prenatal Testing (NIPT) is genetic testing of fetal abnormalities, such as Patau's syndrome, Edward's syndrome, and Down syndrome caused by more than one trisomy pair of 13, 18 and 21 chromosomes, respectively. NIPT screening has a direct benefit in preventing the occurrence of recurrent genetic diseases in families whose first child has a genetic disease. Besides, it can reduce the risk of miscarriage and amniotic fluid drilling, which is the traditional standard method, and reduce medical expenses on the care of patients with genetic diseases. Cost avoidance was the expected cost form the treatment of these genetic diseases, which might occur in the future. However, there was no productivity gain from NIPT.

#### Hereditary cardiomyopathy panel testing using NGS technology

Hereditary cardiomyopathy panel testing using NGS technology will help provide the information on the family specific mutations and the risk of family members who are considered at high risk for developing heart disease due to genes associated with myocardial infarction (MI) and major arteries (cardiomyopathy). The most common type is dilated cardiomyopathy [15], which causes the expansion of the heart with abnormal compression of the heart. It is often found with a thickened heart muscle resulting in an increased demand for oxygenation of the heart muscle, which may cause MI or ischemia leading to death. Therefore, if the patient has undergone genetic testing and knew that there was a high risk of heart disease, the genetic disease can be prevented by participating in disease monitoring programs, such as performing an echocardiogram regularly and modifying behaviors to reduce the risk of death from MI. This will lead to the avoidance of the cost of treating MI in the future. Additionally, those without family risks do not need to go into the surveillance programs, which can reduce unnecessary medical expenses. Cost avoidance was calculated by the treatment cost of MI, which can be avoided in the future. We assumed that the treatment for patients with MI would be given throughout their lifetime period. Productivity gain was calculated by the annual income of patients multiplied by the number of life-years gained for those with hereditary cardiomyopathy panel testing as compared with those without testing.

## Results

### Cost assessment

Table 2 shows the total cost of 103 million baht during 2014 to 2018 with an average annual cost of 21 million baht per year involved in the operation of the CMG. Of the total amount, 72% (74 million baht) was funded by the Faculty of Medicine Ramathibodi Hospital, while 28% (29 million baht) was funded by the TCELS under the Ministry of Science and Technology.

**Table 2:** Cost-benefit analysis results.

Cost-benefit analysis results	Provider (CMG) perspective (million baht)	Societal Perspective (million baht)
Total cost	103	103
Total benefit	136	5,994
Net benefit	33	5,891
Benefit-to-cost ratio	1.32	58.24
Return on investment	0.32	57.24

### Benefit assessment

#### Revenues from genetic testing services at the CMG

**Fig 1:**
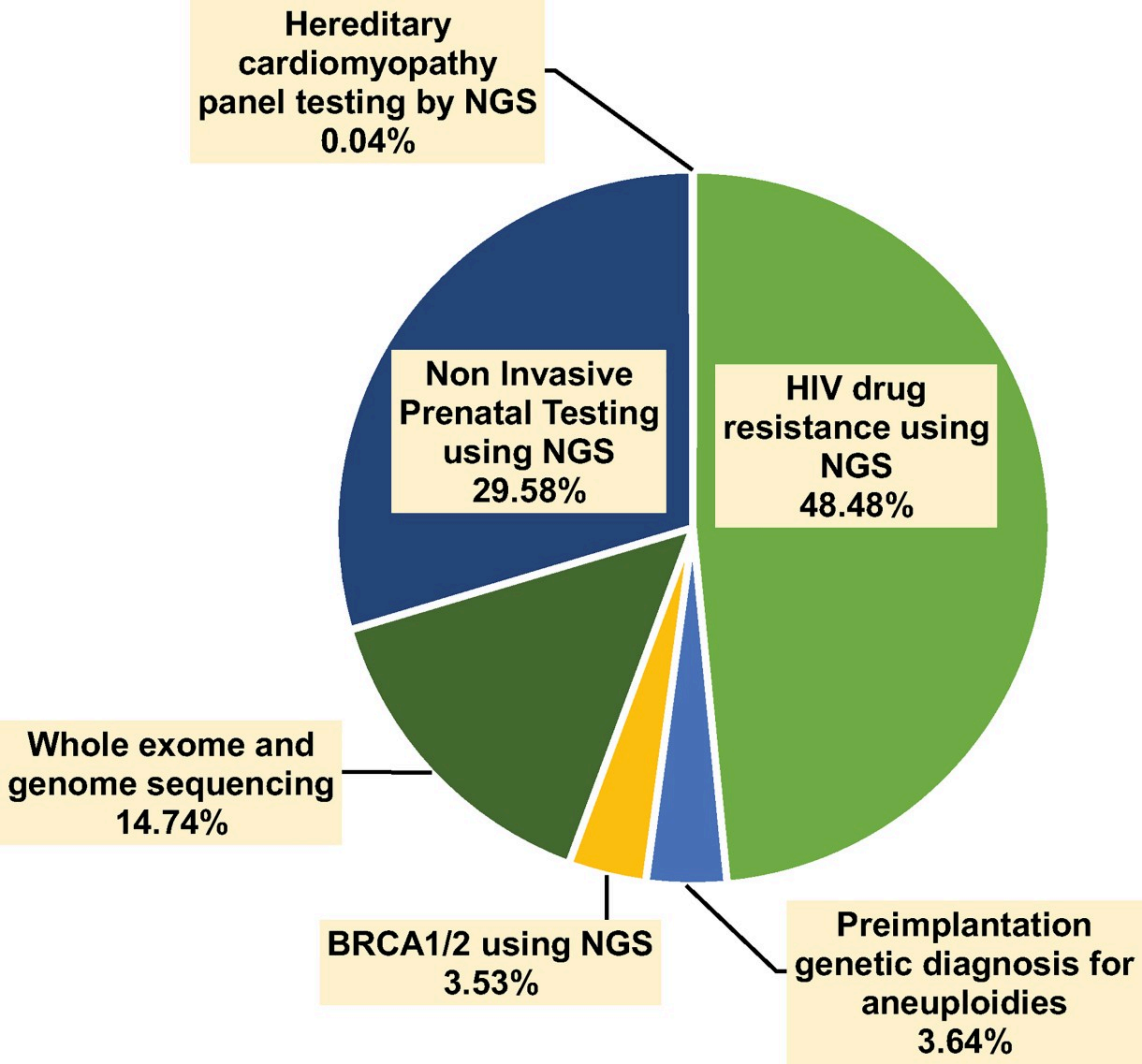
Revenues of the genetic testing services at the CMG during 2014–2018.

Fig 1 presents that the total revenues generated from genetic testing services at the CMG during 2014 to 2018 were approximately 136 million baht with an average annual revenue of 27 million baht. The highest income was generated from genetic testing for HIV drug resistance (48.48%), followed by Thai NIPT (29.58%) and whole exome and genome sequencing (14.74%).

#### Cost-saving from genetic testing services at the CMG compared to the services in overseas

**Fig 2:**
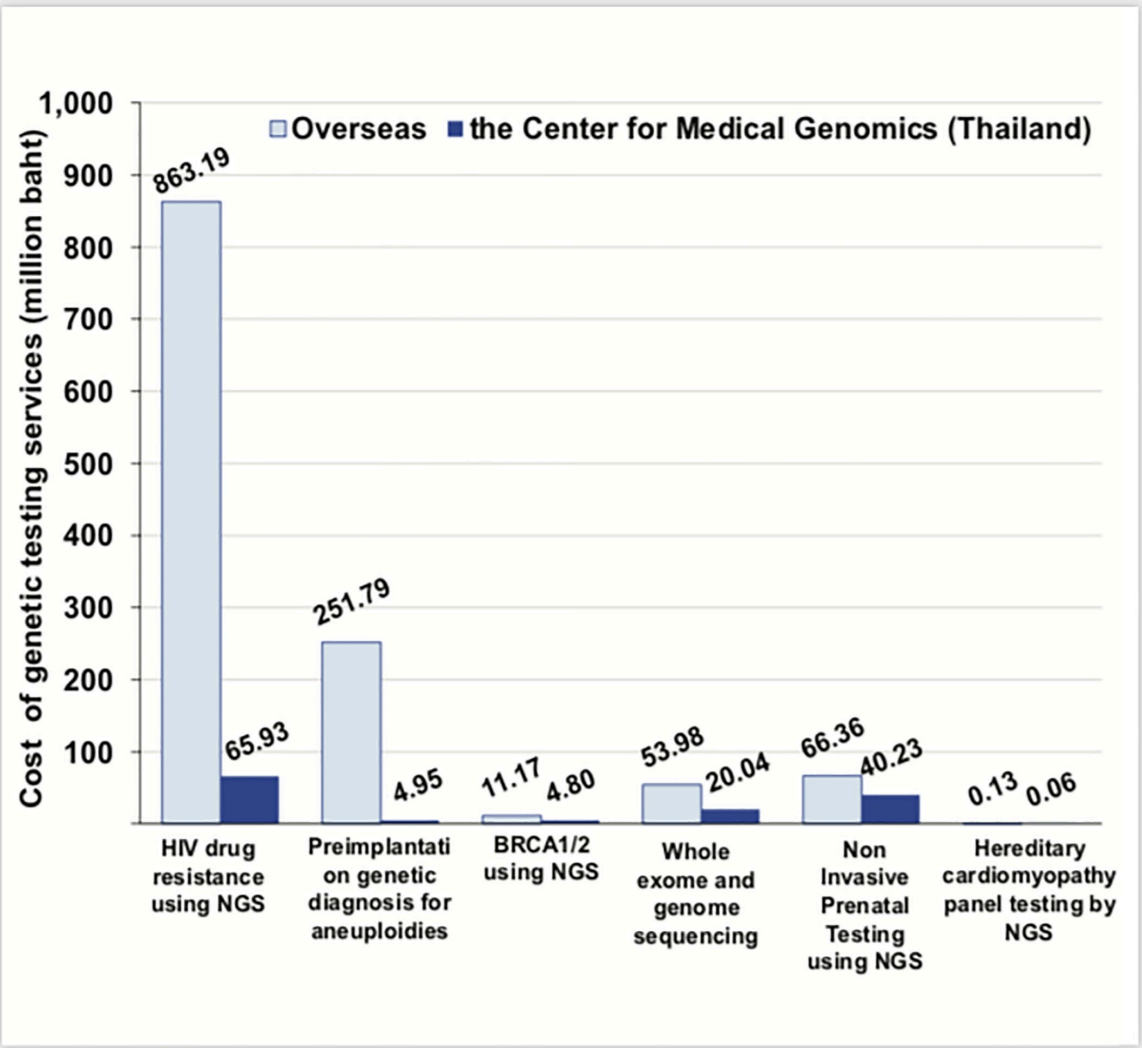
Cost of the genetic testing services provided by agencies overseas and the CMG.

Fig 2 demonstrates that compared to genetic testing services overseas, genetic testing services at CMG during 2014 to 2018 could save 1,110 million baht with an average annual cost-saving of 222 million baht. Genetic testing for HIV drug resistance yielded the highest cost-saving (71.79%), followed by embryo screening before transferring to the uterus using NGS technology (22.23%) and whole exome and genome sequencing (3.06%).

#### Cost avoidance and productivity gain from genetic testing services

The expected cost of unnecessary second-line drugs that could be avoided in the future due to genetic testing for HIV drug resistance was approximately 621 million baht. Moreover, the detection of HIV drug resistance will help select appropriate ARV drugs for HIV patients who do not respond to the first-line regimen. This could prevent death or extend the expected life expectancy resulting in a productivity gain of 2,964 million baht. Furthermore, preimplantation genetic testing for aneuploidies resulted in the cost that can be avoided in the future from the treatment for genetic diseases caused by chromosomal abnormalities of 960 million baht. The top five diseases with cost avoidance from the treatment of genetic diseases were Gaucher's syndrome (500 million baht), DiGeorge-Velocardiofacial syndrome (186 million baht), hemophilia B (82 million baht), polycystic kidney disease (63 million baht), and osteogenesis imperfecta types I-IV (58 million baht).

Screening for BRCA1/2 gene mutations in breast and ovarian cancer using NGS technology could avoid a treatment cost of 42 million baht for patients with advanced breast cancer (37 million baht) or advanced ovarian cancer (5 million baht). Moreover, the detection of BRCA1/2 gene mutations in breast and ovarian cancer with NGS technology could reduce the risk of death due to early detection of the diseases and extend the expected life expectancy leading to a total productivity gain (51 million baht) for breast (44 million baht) or ovarian (7 million baht) cancer patients. Providing Thai NIPT yielded a total cost avoidance of 77 million baht from the treatment of genetic diseases or chromosomal abnormalities, e.g., Down syndrome (48 million baht), and Patau’s syndrome and Edward’s syndrome (29 million baht). Hereditary cardiomyopathy panel testing by NGS could help reduce the risk of death due to cardiomyopathy or extend the expected life expectancy which could generate an additional income of 130,360 baht.

Of all genetic testing services at the CMG, the total cost avoidance of genetic testing for preimplantation genetic testing for aneuploidies was the highest (960 million baht), followed by HIV drug resistance (621 million baht), and Thai NIPT (76 million baht). The total productivity gain of genetic testing for HIV drug resistance was the highest (2,964 million baht), followed by BRCA1/2 gene mutation screening for breast and ovarian cancers (51 million baht).

**Fig 3:**
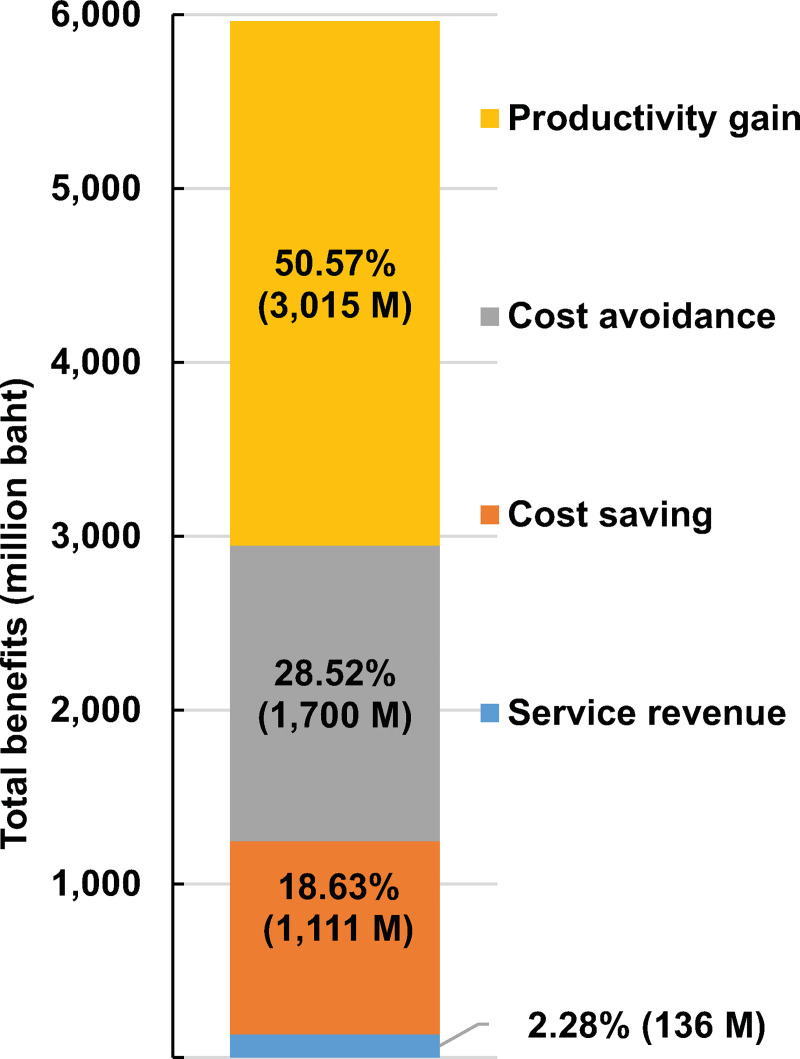
The total benefit of all genetic testing services at the CMG during 2014–2018 by types of benefit (total value 5,962 million baht).

Fig 3 shows that the total benefit of all genetic testing services at the CMG during 2014 to 2018 was 5,962 million baht with an average annual benefit of 1,192 million baht, accounting for productivity gain (50.57%), cost avoidance (28.52%), cost-saving from the tests being sent abroad (18.63%) and revenues from the genetic testing services (2.28%). Of all genetic testing services at the CMG, screening for HIV drug resistance yielded the highest total benefit (74.61%) due to high revenues (48.48%), cost-saving (71.79%), cost avoidance (36.52%) and productivity gain (98.30%).

### Cost-benefit analysis

The results of cost-benefit analysis of genetic testing services provided by the CMG during 2014 to 2018 showed that the total cost of CMG establishment was 103 million baht; whereas the total benefit was 5,994 million baht (Table 2). Therefore, according to a societal perspective, the net benefit, benefit-to-cost ratio and benefit-to-cost ratio were 5,891 million baht, 58.24, and 57.24, respectively. However, based on CMG’s perspective, which considered only the revenue generated by the CMG, a net benefit was decreased to 33 million baht, the benefit-to-cost ratio was 1.32, and the return on investment was 0.32 times.

## Discussion

Over the past decades, significant advances in the field of medical genetic testing have been observed in Thailand [16]. However, there has been limited study related to the economic impact of medical genetic testing services in Thailand, which is of much interest to policy makers. Our study has been the first to investigate the costs and benefits of genetic testing services provided by the CMG during 2014 to 2018 based on provider and societal perspectives in Thailand, which is a developing country where healthcare resources for medical genomics are limited.

The results suggested that the provision of genetic testing services by the CMG would gain more benefit than cost in a Thai society. Furthermore, of all types of benefit from genetic testing services, productivity gain was the highest proportion, followed by cost avoidance, cost-saving, and revenues. In addition to generating revenues for the CMG, genetic testing services can help reduce costs and budgets for the Thai government and society since there is no need to use overseas services, which are more expensive. Moreover, genetic testing could result in treatment cost avoidance in the future and productivity gain in society due to its life prolongation for high-risk people. When the total benefit was classified by the type of genetic testing, genetic testing for HIV drug resistance yielded the highest benefit. It can be explained by the fact that the CMG provided the highest number of genetic testing services for HIV drug resistance since the cost of service is covered by all health insurance schemes in the country. This implies that all HIV patients can get access to the service [17]. Nevertheless, it has been challenging to incorporate medical genetic testing into the reimbursement health benefit package under the universal coverage scheme (UCS), which covers majority of the Thai population (80%) [10].

On the other hand, based on the CMG’s perspective, which considered only the revenue as the benefit, it is noted that genetic testing services would gain little benefit over cost as they are under a governmental, university-based hospital where high mark-up prices are usually not observed. Our study suggested that the establishment of CMG as a medical genomics setting would be a good value for money and worth for investment in Thailand. Furthermore, these findings could be used to support policy decision-making on the establishment of a medical genomics setting to provide genetic testing services in other developing countries, where there is tendency to implement under the UCS but may have resource constraints on health care services.

Nevertheless, the implementation of a medical genomics setting in the national health policy depends on many factors, such as socio-economic status, medical genetic infrastructure, international collaboration, law and regulation, cultural perspective and medical care ecology of the country [5, 6, 18]. In terms of socio-economic status and medical genetic infrastructure, lack of budget and public awareness on genetic testing, lack of government support and genetic testing services have been issues on the accessibility of such services. Consequently, these could hinder the implementation of a medical genomics setting into the national health policy, especially among low- and middle-income countries (LMICs) in Asia [18]. Correspondingly, a research project conducted by the Organization for Economic Co-operation and Development (OECD) and the UK-based Economic and Social Research Council (ESRC) Genomic network on Personalised Medicine and Global Public Health revealed that the application of genomics for personalized medicine has been predominantly increasing, however few successful implementations were established. In addition, high-income countries were usually less dependent on international collaborations for personalized medicine, while LMICs depended much more on international collaborations from high-income countries [19].

Interestingly, among the medical genomic applications for personalized medicine, genetic screening and testing for genetic diseases and prenatal screening were widely applied, as they were necessary to focus not only on survival, but also on health, growth and development of newborns [20]. Therefore, it was recommended that research studies related to medical genomics that could reduce neonatal mortality, morbidity and long-term impairment should be prioritized. Also, the government and private sectors should financially support the conduct of such studies [20]. The impact of law and regulation as well as cultural perspective on the implementation of the medical genomics setting could be evidently seen in China, where a “one family, one child” policy and a family planning law that allowed to have only one child were implemented. Later, the “1.5 child” policy, which permitted a married couple whose first child was a girl to have a second child after, was carried out in 1984 since most Chinese preferred sons rather than daughters based on the Chinese culture perspective. The law stated that pregnant women should have a legal abortion if prenatal screening test shows that the fetus has serious hereditary diseases [21]. Therefore, these factors led to a high demand on prenatal screening in China. Although the Chinese government has provided more services in prenatal screening, not everyone could get an access to these services because the coverage rates of prenatal screening and screening fees varied across the provinces [21]. Thus, medical care ecology investigating the pattern of healthcare consumption and utilization of available healthcare resources in China was explored to understand socio-demographic characteristics of patients receiving care in different settings [6]. Since medical genetic testing is an expensive service and difficult to get access based on patients’ viewpoint, studies on medical care ecology of medical genetic testing should be performed to provide useful information on the implementation of medical genetic testing services for policy decision-makers [6].

Notably, the limitations of this study were needed to be addressed. First, due to the lack of data available in Thailand, data on some parameters were obtained from published literatures in international countries. Specifically, to calculate cost-saving from the tests being sent abroad, their prices were obtained from literature reviews or price information available on the websites of genetic testing agencies in the United States, United Kingdom, or Canada as we could not find the price information disclosed from the agencies that provide genetic testing services in Asian countries. It should be highlighted that this could lead to an overestimated cost-saving, since most people usually did not pay the advertised price which could be negotiated and discounted. In addition, a price reduction under a service provider agreement could result in a decrease in benefit to the government. Therefore, future studies using the actual costs found from Asian agencies should be further investigated. Second, medical expenses and productivity gain were calculated throughout the patient's lifespan as we assumed that they would receive treatment and could earn income for the rest of their lives. Consequently, this might lead to an overestimation of the benefit. Moreover, providing NIPT to pregnant women could facilitate a productivity benefit by not having to spend time for caring a child with genetic disorders. Nevertheless, productivity gain was not accounted for, since it was beyond the scope of this study. Third, we did not take into account the costs resulting from other diseases, such as chronic non-communicable diseases, which might occur as patients would have longer life expectancy due to receiving genetic testing services. Therefore, this may lead to an overestimation of the benefit. Fourth, we included the total cost of the operation at the CMG as the cost for managing genetic testing services. However, this cost may be overestimated since the CMG also provides other services, such as training, arranging academic conferences, and networking with other national and international organizations. Fifth, we estimated the economic impact of the provision of genetic testing services at the CMG only, which may not be a representative of all genetic testing centers in Thailand. However, we expect that the benefits would be higher than the costs if we estimate for all governmental genetic testing settings. Lastly, apart from the economic impact of medical genetic services, other impacts based on the perspectives of patients, healthcare personnel and policy makers were not considered considering that the CMG has performed research and provided genetic knowledge and techniques through training and academic conferences for medical personnel, scholars, researchers in both domestic and international organizations. This would result in academic advancement, which could affect patient care, realize the importance of genetic research, and drive health policy. Thus, further research is warranted to explore these impacts as these can demonstrate the usefulness of the services, in terms of clinical outcomes and mental health of service recipients, healthcare decision-making, research, and knowledge dissemination.

## Conclusions

Our study suggested that the provision of genetic testing services at the CMG would gain more benefit than cost in Thai society. It highlighted that the establishment of the CMG would provide good value for money and would be worth for the investment in Thailand. These findings could be used to support policy decision-making on the establishment of medical genomics settings to provide medical genetic testing services not only in Thailand but also in other developing countries.

## Supporting information

S1 Table Unit price of gene testing services at the CMG and agencies overseas. (DOCX)

S2 Table Variables used to calculate cost avoidance and productivity gain. (DOCX)
