# Supplementary material for: Economic impact of medical genetic testing on clinical applications in Thailand
Source: PLoS One. 2020 Dec 18;15(12):e0243934. doi: 10.1371/journal.pone.0243934 (PMC7748141; doi:10.1371/journal.pone.0243934)
Supplement: S1 Table — (DOCX) [file pone.0243934.s001.docx]

**Economic Impact of Medical Genetic Testing on Clinical Applications in Thailand**

Jiraphun Jittikoon, Sermsiri Sangroongruangsri, Montarat Thavorncharoensap, Natthakan Chitpim, Usa Chaikledkaew*

**S1 Table** **Unit price of gene testing services at the CMG and agencies overseas.**

| **Medical genetic testing services** | **Unit price (baht)** | **References** |
| --- | --- | --- |
| **HIV drug resistance using NGS** | |  |
| HIV drug resistance in Thailand | 6,000 | Center of Medical Genomics, 2018[1] |
| HIV drug resistance in the US | 78,551 | Inzaule, 2016[2] |
| **Preimplantation genetic diagnosis for aneuploidies** | |  |
| Multiplex SNP genotyping in Thailand | 900 | Center of Medical Genomics, 2018[1] |
| Multiplex SNP genotyping in the US | 52,922 | The University of Arizona Genetics Core [3] |
| Low pass whole genome sequencing – WGA in Thailand | 9,800 | Center of Medical Genomics, 2018[1] |
| Low pass whole genome sequencing – WGA in Canada | 497,607 | Weymann, 2017[4] |
| Mitochondrial DNA analysis – Encephalomyopathy in Thailand | 3,250 | Center of Medical Genomics, 2018[1] |
| Mitochondrial DNA analysis – Encephalomyopathy in the US | 21,158 | Prevention Genetics [5] |
| Prenatal diagnosis using direct mutation analysis in Thailand | 3,750 | Center of Medical Genomics, 2018[1] |
| Prenatal diagnosis using direct mutation analysis in the US | 32,729 | Genetics, 2018[6] |
| **BRCA1/2 using NGS** |  |  |
| HBOC panel testing – blood by NGS in Thailand | 23,600 | Center of Medical Genomics, 2018[1] |
| HBOC panel testing – blood by NGS in the US | 39,341 | Genetics, 2018[6] |
| Real time PCR – Others at CMG | 2,650 | Center of Medical Genomics, 2018[1] |
| Real time PCR – Others in the US | 10,745 | University of Nebraska Medical Center, 2018[7] |
| **Whole exome and genome sequencing** |  |  |
| Whole gene mutation screening – Others by NGS in Thailand | 42,500 | Center of Medical Genomics, 2018[1] |
| Whole gene mutation screening – Others by NGS in the US | 95,213 | The University of Arizona Genetics Core, 2018[8] |
| Multiple coding region sequencing by NGS in Thailand | 40,600 | Center of Medical Genomics, 2018[1] |
| Multiple coding region sequencing by NGS in the US | 95,213 | The University of Arizona Genetics Core, 2018[8] |
| Targeted gene sequencing analysis by NGS in Thailand | 6,200 | Center of Medical Genomics, 2018[1] |
| Targeted gene sequencing analysis by NGS in the US | 20,828 | Boston University Medical Campus: Microarray and Sequencing Resource, 2018[9] |
| Whole gene sequencing – Others in Thailand | 5,950 | Center of Medical Genomics, 2018[1] |
| Whole gene sequencing – Others in the US | 33,027 | Dante Labs, 2018[10] |
| Next generation viral sequencing in Thailand | 27,000 | Center of Medical Genomics, 2018[1] |
| Next generation viral sequencing in the UK | 87,071 | Moleerergpoom, 2007[11] |
| **Non Invasive Prenatal Testing (NIPT) using NGS** | |  |
| IONA NIPT by NGS in Thailand | 17,000 | Center of Medical Genomics, 2018[1] |
| IONA NIPT by NGS in the UK | 17,492 | Antenatal Clinic Saint Mary's Hospital, 2018[12] |
| Thai NIPT by NGS in Thailand | 10,600 | Center of Medical Genomics, 2018[1] |
| Thai NIPT by NGS in the UK | 17,492 | Antenatal Clinic Saint Mary's Hospital, 2018[12] |
| **Hereditary cardiomyopathy panel testing using NGS technology** | | |
| Hereditary cardiomyopathy panel testing by NGS in Thailand | 30,500 | Center of Medical Genomics, 2018[1] |
| Hereditary cardiomyopathy panel testing by NGS in the US | 65,459 | Genetics, 2018 [6] |

**References**

1. Center of Medical Genomics. Price list for each medical genetic testing service. Bangkok, Thailand: Center of Medical Genomics, Faculty of Medicine Ramathibodi Hospital; 2018.

2. Inzaule S, Ondoa P, Peter T, Mugyenyi P, Stevens W, Rinke de Wit T, et al. Affordable HIV drug-resistance testing for monitoring of antiretroviral therapy in sub-Saharan Africa. Lancet Infect Dis. 2016;16(11):e267-e75.

3. The University of Arizona Genetics Core. SNP genotyping - mass array [cited 2018 November 15]. Available from: <https://uagc.arl.arizona.edu/snp-genotyping-mass-array>.

4. Weymann D, Laskin J, Roscoe R, Schrader K, Chia S, Yip S, et al. The cost and cost trajectory of whole‐genome analysis guiding treatment of patients with advanced cancers. Mol Genet Genomic Med. 2017;5(3):251–60.

5. Prevention Genetics. SUCLG1-related encephalomyopathic form of mitochondrial DNA depletion syndrome via the SUCLG1 gene [cited 2018 November 15]. Available from: <https://www.preventiongenetics.com/searchTests.php?val=SUCLG1-&sel=test>.

6. Genetics P. Clinical DNA test menu: January 2018 price by gene 2018 [cited 2018 November 15]. Available from: <https://www.preventiongenetics.com/documents/Pricelist_By_Gene.pdf>.

7. University of Nebraska Medical Center. Gene expression analysis (QPCR) pricing 2018 [cited 2018 November 15]. Available from: <https://www.unmc.edu/vcr/cores/vcr-cores/ecf/pricing/gene-expression-pricing.html>.

8. The University of Arizona Genetics Core. ILLUMINA HISEQ 2500 2018 [cited 2018 November 15]. Available from: <https://uagc.arl.arizona.edu/illumina-hiseq-2500>.

9. Boston University Medical Campus: Microarray and Sequencing Resource. IonTorrent PGM & Proton Next Gen Sequencing 2018 [cited 2018 November 23]. Available from: <http://www.bumc.bu.edu/microarray/pricing/ion-torrent-sequencing/>.

10. Dante Labs. Whole genome sequencing with mtDNA 2018 [cited 2018 November 15]. Available from: <https://www.dantelabs.com/products/whole-genome-sequencing>.

11. Moleerergpoom W, Kanjanavanit R, Jintapakorn W, Sritara P. Costs of payment in Thai acute coronary syndrome patients. J Med Assoc Thai. 2007;90:21-31.

12. Antenatal Clinic Saint Mary's Hospital. The IONA test Oxford: Antenatal Clinic Saint Mary's Hospital,; 2018 [cited 2018 November 15]. Available from: <http://www.trafford.nhs.uk/media/1703971/n4090%20iona%20lft%20for%20pregnant%20women%20st%20marys%20mkt031.pdf>.
