# Supplementary material for: Economic impact of medical genetic testing on clinical applications in Thailand
Source: PLoS One. 2020 Dec 18;15(12):e0243934. doi: 10.1371/journal.pone.0243934 (PMC7748141; doi:10.1371/journal.pone.0243934)
Supplement: S2 Table — (DOCX) [file pone.0243934.s002.docx]

**Economic Impact of Medical Genetic Testing on Clinical Applications in Thailand**

Jiraphun Jittikoon, Sermsiri Sangroongruangsri, Montarat Thavorncharoensap, Natthakan Chitpim, Usa Chaikledkaew*

**S2 Table** **Variables used to calculate cost avoidance and productivity gain.**

| **Parameter** | **Value** | **References** |
| --- | --- | --- |
| **HIV drug resistance** |  |  |
| Probability of HIV patients who do not respond to the first line ARV regimen and have HIV drug resistant gene | 0.935 | Sungkanuparph, 2007[1] |
| Annual cost of first line ARV drugs per person (baht) | 12,216 | Sungkanuparph, 2007[1] |
| Annual cost of second line ARV drugs per person (baht) | 28,963 | Sungkanuparph, 2007[1] |
| Life years gained for HIV patients with baseline CD4 ≥ 350 cells/mm3 starting conventional ARV treatment at aged 20 years (years) | 51.9 | Teeraananchai, 2017[2] |
| Life years gained for HIV patients who did not respond to the first line ARV regimen after receiving genetic testing for HIV drug resistant (years) | 1.2 | Sax, 2005[3] |
| Annual income per person (baht) | 224,759 | The World Bank, 2017[4] |
| Number of people receiving the test for HIV drug resistance | 10,989 | Center of Medical Genomics, 2018[5] |
| **Preimplantation genetic testing for aneuploidies** |  |  |
| Probability of people receiving preimplantation genetic testing and having positive test | 0.28 | Center of Medical Genomics, 2018[5] |
| Probability of having beta-thalassemia | 0.03 | Center of Medical Genomics, 2018[5] |
| Probability of having Gaucher's Syndrome | 0.13 | Center of Medical Genomics, 2018[5] |
| Probability of having DiGeorge-Velocardiofacial syndrome | 0.09 | Center of Medical Genomics, 2018[5] |
| Probability of having polycystic kidney disease | 0.07 | Center of Medical Genomics, 2018[5] |
| Probability of having neurofibromatosis type 1 | 0.05 | Center of Medical Genomics, 2018[5] |
| Probability of having hemophilia B | 0.04 | Center of Medical Genomics, 2018[5] |
| Probability of having spinal muscular atrophy | 0.04 | Center of Medical Genomics, 2018[5] |
| Probability of having Duchenne muscular dystrophy | 0.01 | Center of Medical Genomics, 2018[5] |
| Probability of having Marfan syndrome | 0.01 | Center of Medical Genomics, 2018[5] |
| Probability of having glutaric acidemia IIB | 0.01 | Center of Medical Genomics, 2018[5] |
| Probability of having osteogenesis imperfecta types I-IV | 0.10 | Center of Medical Genomics, 2018[5] |
| Probability of having Hallervorden-Spatz syndrome | 0.08 | Center of Medical Genomics, 2018[5] |
| Probability of having Ellis-van Creveld syndrome | 0.01 | Center of Medical Genomics, 2018[5] |
| Probability of having multiple congenital anomalies-hypotonia-seizures syndrome | 0.01 | Center of Medical Genomics, 2018[5] |
| Cost of treatment at neonatal intensive care unit (baht per day) | 12,312 | Songwai, 2017[6] |
| Annual cost of treatment for patients with beta-thalassemia (baht) | 32,788 | Riewpaiboon, 2010[7] |
| Life expectancy of patients with beta-thalassemia (years) | 20 | Zamani, 2015[8] |
| Annual cost of treatment for patients with Gaucher's Syndrome (baht) | 2,500,000 | Expert opinion |
| Life expectancy of patients with Gaucher's Syndrome (years) | 20 | Expert opinion |
| Lifetime cost of treatment for patients with DiGeorge-Velocardiofacial syndrome (baht) | 26,501,933 | Benn, 2017[9] |
| Life expectancy of patients with DiGeorge-Velocardiofacial syndrome (years) | 20 | Benn, 2017[9] |
| Lifetime cost of treatment for patients with polycystic kidney disease (baht) | 12,599,256 | Eriksson, 2017[10] |
| Life expectancy of patients with polycystic kidney disease (years) | 60 | Perrone, 2001[11] |
| Annual cost of treatment for patients with neurofibromatosis type 1 (baht) | 39,664 | Wolkenstein, 2000 [12] |
| Life expectancy of patients with neurofibromatosis type 1 (years) | 60 | Expert opinion |
| Annual cost of treatment for patients with hemophilia B (baht) | 27,484,810 | Pattanaprateep, 2014[13] |
| Life expectancy of patients with hemophilia B (years) | 63 | Darby, 2007[14] |
| Annual cost of treatment for patients with spinal muscular atrophy (baht) | 7,879,807 | Expert opinion |
| Life expectancy of patients with spinal muscular atrophy (years) | 2 | Expert opinion |
| Annual cost of treatment for patients with Duchenne muscular dystrophy (baht) | 57,188 | Thayer, 2017[15] |
| Life expectancy of patients with Duchenne muscular dystrophy (years) | 19 | Thayer, 2017[15] |
| Annual cost of treatment for patients with Marfan syndrome (baht) | 216,384 | Achelrod, 2014 [16] |
| Life expectancy of patients with Marfan syndrome (years) | 60 | Achelrod, 2014[16] |
| Lifetime cost of treatment for patients with glutaric acidemia IIB (baht) | 4,493,953 | Expert opinion |
| Life expectancy of patients with glutaric acidemia IIB (year) | 1 | Cipriano, 2007[17] |
| Lifetime cost of treatment for patients with osteogenesis imperfecta types I-IV (baht) | 7,266,196 | Expert opinion |
| Life expectancy of patients with osteogenesis imperfecta types I-IV (years) | 50 | Expert opinion |
| Lifetime cost of treatment for patients with Hallervorden-Spatz syndrome (baht) | 2,216,196 | Expert opinion |
| Life expectancy of patients with Hallervorden-Spatz syndrome (year) | 0.5 | Expert opinion |
| Lifetime cost of treatment for patients with Ellis-van Creveld syndrome (baht) | 123,122 | Expert opinion |
| Life expectancy of patients with Ellis-van Creveld syndrome (days) | 10 | Expert opinion |
| Lifetime cost of treatment for patients with multiple congenital anomalies-hypotonia-seizures syndrome (baht) | 4,493,953 | Expert opinion |
| Life expectancy of patients with multiple congenital anomalies-hypotonia-seizures syndrome (year) | 1 | Expert opinion |
| **BRCA1/2 using NGS technology** |  |  |
| Probability of people receiving BRCA1/2 and having positive test | 0.14 | Nelson, 2013[18] |
| Probability of having breast cancer with positive test | 0.46 | Nelson, 2013[18] |
| Probability of having ovarian cancer with positive test | 0.17 | Nelson, 2013[18] |
| Annual cost of treatment for patients with advanced-stage breast cancer (baht) | 767,432 | Kongsakon, 201[19] |
| Life expectancy for patients with advanced-stage breast cancer (years) | 1.12 | Laohavinij, 2017[20] |
| Annual cost of treatment for patients with advanced-stage ovarian cancer (baht) | 654,936 | Luealon, 2016[21] |
| Life expectancy for patients with advanced-stage ovarian cancer (years) | 0.51 | Luealon, 2016[21] |
| Life years gained for breast cancer patients receiving BRCA1/2 genetic testing (years) | 0.3 | Lim, 2018[22] |
| Life years gained for ovarian cancer patients if receiving BRCA1/2 genetic testing (years) | 0.05 | Kwon, 2010 [23] |
| Number of patients receiving BRCA1/2 genetic testing | 648 | Center of Medical Genomics, 2018[5] |
| **Non Invasive Prenatal Testing (NIPT)** |  |  |
| Probability of having Patau’s syndrome | 0.0016 | Center of Medical Genomics, 2018[5] |
| Probability of having Edward’s syndrome | 0.0042 | Center of Medical Genomics, 2018[5] |
| Probability of having Down’s syndrome | 0.0071 | Center of Medical Genomics, 2018[5] |
| Lifetime cost of treatment for patients with Down’s syndrome (baht) | 1,788,846 | Patanapesaj J, 2011[24] |
| Life expectancy for patients with Down’s syndrome (years) | 50 | Jitprapan, 2014[25] |
| Lifetime cost of treatment for patients with Patau’s syndrome and Edward’s syndrome (baht) | 1,314,431 | Walker, 2015[26] |
| Life expectancy for patients Patau’s syndrome and Edward’s syndrome (year) | 1 | Peroos, 2012[27] |
| **Hereditary cardiomyopathy panel testing by NGS** |  |  |
| Probability of people receiving hereditary cardiomyopathy panel testing and having a positive test | 0.20 | Center of Medical Genomics, 2018[5] |
| Probability of having cardiomyopathy with positive test | 0.4 | Sweet, 2015[28] |
| Lifetime cost of treatment for patients with cardiomyopathy (baht) | 810,311 | Saokaew, 2014[29] |
| Life years gained for patients with cardiomyopathy if receiving hereditary cardiomyopathy panel testing (years) | 0.29 | Perez, 2011[30] |
| Number of patients receiving hereditary cardiomyopathy panel testing | 2 | Center of Medical Genomics, 2018[5] |

**References**

1. Sungkanuparph S, Manosuthi W, Kiertiburanakul S, Piyavong B, Chumpathat N, Chantratita W. Options for a second-line antiretroviral regimen for HIV type 1-infected patients whose initial regimen of a fixed-dose combination of stavudine, lamivudine, and nevirapine fails. Clin Infect Dis. 2007;44(3):447-52.

2. Teeraananchai S, Chaivooth S, Kerr SJ, Bhakeecheep S, Avihingsanon A, Teeraratkul A, et al. Life expectancy after initiation of combination antiretroviral therapy in Thailand. Antivir Ther. 2017;22(5):393-402.

3. Sax P, Islam R, Walensky R, Losina E, Weinstein M, Goldie S, et al. Should Resistance Testing Be Performed for Treatment-Naive HIV-Infected Patients? A Cost-Effectiveness Analysis. Clin Infect Dis. 2005;41(9):1316–23.

4. The World Bank. GNI per capita, Atlas method (current US$) 2017 [cited 2018 November 20]. Available from: <https://data.worldbank.org/indicator/ny.gnp.pcap.cd>.

5. Center of Medical Genomics. Data on Genetic testing services at the Center of Medical Genomics during 2014-2018. Bangkok, Thailand: Center of Medical Genomics, Faculty of Medicine Ramathibodi Hospital; 2018.

6. Songwai S-N. Unit cost analysis of Fang hospital, Chiang Mai province. Chiangrai Medical Journal. 2017;9(1/2560):133-46.

7. Riewpaiboon A, Nuchprayoon I, Torcharus K, Indaratna K, Thavorncharoensap M, Ubol B-O. Economic burden of beta-thalassemia/Hb E and beta-thalassemia major in Thai children. BMC Res Notes. 2010;3:29.

8. Zamani R, Khazaei S, Rezaeian S. Survival analysis and its associated factors of Beta thalassemia major in hamadan province. IJMS. 2015;40(3):233-9.

9. Benn P, Iyengar S, Crowley TB, Zackai EH, Burrows EK, Moshkevich S, et al. Pediatric healthcare costs for patients with 22q11.2 deletion syndrome. Mol Genet Genomic Med. 2017;5(6):631-8.

10. Eriksson D, Karlsson L, Eklund O, Dieperink H, Honkanen E, Melin J, et al. Real-world costs of autosomal dominant polycystic kidney disease in the Nordics. BMC Health Serv Res. 2017;17(1):560.

11. Perrone RD, Ruthazer R, Terrin NC. Survival after end-stage renal disease in autosomal dominant polycystic kidney disease: contribution of extrarenal complications to mortality. Am J Kidney Dis. 2001;38(4):777-84.

12. Wolkenstein P, Durand-Zaleski I, Moreno JC, Zeller J, Hemery F, Revuz J. Cost evaluation of the medical management of neurofibromatosis 1: a prospective study on 201 patients. Br J Dermatol. 2000;142(6):1166-70.

13. Pattanaprateep O, Chuansumrit A, Kongsakon R. Cost-Utility Analysis of Home-Based Care for Treatment of Thai Hemophilia A and B. Value Health Reg Issues. 2014;3:73-8.

14. Darby SC, Kan SW, Spooner RJ, Giangrande PLF, Hill FGH, Hay CRM, et al. Mortality rates, life expectancy, and causes of death in people with hemophilia A or B in the United Kingdom who were not infected with HIV. Blood. 2007;110(3):815.

15. Thayer S, Bell C, McDonald CM. The Direct Cost of Managing a Rare Disease: Assessing Medical and Pharmacy Costs Associated with Duchenne Muscular Dystrophy in the United States. J Manag Care Spec Pharm. 2017;23(6):633-41.

16. Achelrod D, Blankart CR, Linder R, von Kodolitsch Y, Stargardt T. The economic impact of Marfan syndrome: a non-experimental, retrospective, population-based matched cohort study. Orphanet journal of rare diseases. 2014;9:90.

17. Cipriano LE, Rupar CA, Zaric GS. The Cost-Effectiveness of Expanding Newborn Screening for up to 21 Inherited Metabolic Disorders Using Tandem Mass Spectrometry: Results from a Decision-Analytic Model. Value in Health. 2007;10(2):83-97.

18. Nelson H, Fu R, Goddard K, Mitchell J, Okinaka-Hu L, Pappas M, et al. Risk Assessment, Genetic Counseling, and Genetic Testing for BRCA-Related Cancer: Systematic Review to Update the U.S. Preventive Services Task Force Recommendation. Rockville (MD): Agency for Healthcare Research and Quality (US); 2013.

19. Kongsakon R, Lochid-amnuay S, Kapol N, Wiratkapun C. Economic Evaluation of Trastuzumab for Treatment of Breast Cancer in Thailand. 2012.

20. Laohavinij S, Paul V, Maneenil K. Survival and Prognostic Factors of Metastatic Breast Cancer. J Med Assoc Thai. 2017;100:S16-26.

21. Luealon P, Khempech N, Vasuratna A, Hanvoravongchai P, Havanond P. Cost effectiveness analysis of different management strategies between best supportive care and second-line chemotherapy for platinum-resistant or refractory ovarian cancer. Asian Pac J Cancer Prev. 2016;17(2):799-805.

22. Lim K, Yoon S, Mohd TN, Shabaruddin F, Dahlui M, Woo Y, et al. s BRCA Mutation Testing Cost Effective for Early Stage Breast Cancer Patients Compared to Routine Clinical Surveillance? The Case of an Upper Middle-Income Country in Asia. Appl Health Econ Health Policy. 2018;16(3):395-406.

23. Kwon J, Daniels M, Sun C, Lu K. Preventing future cancers by testing women with ovarian cancer for BRCA mutations. J Clin Oncol. 2010;28:675–82.

24. Patanapesaj J, Tanmookayakul U, Teerawatananon Y. Cost-benefit analysis of prenatal screening and diagnosis for Down syndrome in Thailand. HITAP. 2011.

25. Jitprapan N. Prevention and Care of Children with Down Syndrome. ThaiJO. 2014;1(3 ).

26. Walker BS, Nelson RE, Jackson BR, Grenache DG, Ashwood ER, Schmidt RL. A Cost-Effectiveness Analysis of First Trimester Non-Invasive Prenatal Screening for Fetal Trisomies in the United States. PLOS ONE. 2015;10(7):e0131402.

27. Peroos S, Forsythe E, Pugh JH, Arthur-Farraj P, Hodes D. Longevity and Patau syndrome: what determines survival? BMJ case reports. 2012;2012:bcr0620114381.

28. Sweet M, Taylor M, Mestroni L. Diagnosis, prevalence, and screening of familial dilated cardiomyopathy. Expert Opin Orphan Drugs. 2015;3(8):869–76.

29. Saokaew S, Hanvoravongchai P. Cost-effectiveness of thrombolysis service for patient with ST segment elevation myocardial infarction in primary care hospital Nonthaburi. Nonthaburi, Thailand: Health Systems Research Institute; 2014.

30. Perez M, Kumarasamy N, Owens D, Wang P, Hlatky M. Cost-effectiveness of genetic testing in family members of patients with long-QT syndrome. Circ Cardiovasc Qual Outcomes. 2011;4(1):76-84.
